# Spiritual Well-Being among Medical and Nonmedical Science Students

**DOI:** 10.1155/2021/6614961

**Published:** 2021-04-24

**Authors:** Mahboobeh Maazallahi, Asma Ghonchepour, Mostafa Sohrabi, Zakiyeh Golestani, Peiman Parandeh Afshar, Alireza Malakoutikhah, Mahlagha Dehghan

**Affiliations:** ^1^Student Research Committee, School of Nursing and Midwifery, Kerman University of Medical Sciences, Kerman, Iran; ^2^Shiraz University of Medical Sciences, Shiraz, Iran; ^3^Nursing Research Center, Kerman University of Medical Sciences, Kerman, Iran

## Abstract

Spiritual well-being is one dimension of health that provides a person with stability, meaning, fulfillment in life, and self-belief. This study aimed to compare the spiritual well-being among students of Kerman University of Medical Sciences and the Shahid Bahonar University of Kerman. With the demographic questionnaire and 20-item spiritual well-being scale of the “Paloutzian and Ellison” questionnaire, a cross-sectional study was conducted on 626 students of the universities of medical and nonmedical sciences by the quota sampling method in Kerman in 2017-2018. The scores of spiritual well-being and its two components were significantly higher in nonmedical science students (89.83 ± 16.79) than in the medical science students (81.61 ± 15.21) (*p* < 0.05). In addition, one percent of the nonmedical science students had a low level, 67.7% had a moderate level, and 31.3% had a high level of spiritual well-being. 0.3% of the medical science students had a low level, 84% had a moderate level, and 15.7% had a high level of spiritual well-being. Since spirituality is important for the profession of medical science students, it may be necessary to expand this component in their curriculum.

## 1. Introduction

University students are at a critical period in their lives that is characterized by individuation and separation from family, development of new social connections, and increased autonomy and responsibility [1]. People in this period face new challenges in education, social relationships, and other fields. These changes lead to increased levels of stress and adaptive and behavioral problems that can adversely impact their health [2, 3]. Spiritual well-being is considered as a psychological resource of coping that reduces suffering and leads people to consider traumatic situations or disturbing events from a positive perspective [4, 5]. Although health has been defined based on three physical, mental, and social dimensions for decades, Russell and Osman included the spiritual dimension in the definition of health [6].

Spiritual well-being is a unique source that coordinates physical, mental, and social dimensions, and it is characterized by stability in life, peace, adjustment, and harmony, a sense of close relationship with oneself, God, society, and the environment [7]. Spiritual well-being consists of two components of religious well-being and existential well-being. Religious well-being is a sign of communication with God, and existential well-being is the sense of purpose in life, peace, and life satisfaction [8]. Spiritual well-being had an effect on the physical, social, and psychological aspects of an individual's life [9]. University students in this period of life take more responsibility for their health and have greater control over their lifestyle than adolescents. Lifestyle changes can have strong implications on their health and well-being [3]. Furthermore, mental health among university students could be affected by spiritual well-being [10].

Various studies investigated the spiritual well-being of university students. Feizi et al. found that healthcare students had moderate spiritual well-being and showed a positive correlation between happiness and spiritual well-being among healthcare students [11]. Moreover, Alorani and Alradaydeh demonstrated a negative correlation between spiritual well-being, depression, and aggression [8]. Fabbris et al. indicated a significant association between well-being and reduced anxiety levels in nursing students [5]. Mehri et al. found a significant relationship between spiritual well-being and health-promoting behaviors [12]. Moreover, one study in Jordan (2015) showed an association between spiritual well-being and better college adjustment among university students [13]. Abdel-Khalek showed a significant and positive correlation between spiritual well-being and quality of life [14].

The transition from adolescence to the youth period causes young people to face sensitive and critical situations. Concerning the role of spiritual well-being in improving the mental and physical well-being of individuals, it is necessary to improve the spiritual well-being of young people, especially students. Since medical science students and nonmedical science students have different talents and educational backgrounds, and medical science students are expected to be aware of and practice healthy behaviors [3], it is necessary to study their spiritual well-being separately to design and implement different interventions in each of the universities. Therefore, this study aimed to compare the students' spiritual well-being in two Kerman universities of medical sciences and nonmedical sciences.

## 2. Materials and Methods

### 2.1. Study Design and Setting

This comparative cross-sectional study was conducted during the academic year of 2017-2018. All students of Kerman University of Medical Sciences and Shahid Bahonar University of Kerman, Iran, were invited to take part in the study. Kerman is the largest city in the southeast of Iran, which is located in Southwest Asia. There are two central governmental universities in Kerman (Kerman University of Medical Sciences and Shahid Bahonar University of Sciences). In the academic year of 2017-2018, 19089 students were studying in different majors and degrees in these universities. In addition, in Iran, all students in medical and nonmedical universities must pass nearly 6–8 general credits about religious and ethical issues.

### 2.2. Sampling and Sample Size

The sample size was estimated to be 300 in each group by using the mean difference formula. Regarding the probability of dropout in each university, 330 questionnaires were provided to the students by using a convenience sampling method. 313 questionnaires were fully delivered from the University of Medical Sciences (*n* = 4556) and 313 from Shahid Bahonar University of Kerman (*n* = 14,533), and ultimately, they were included in the software (response rate 94.85).

### 2.3. Instrument

The demographic characteristics form and the spiritual well-being scale developed by Palutzian & Ellison were used to measure the spiritual well-being of the students. The first part of the survey was related to the demographic data of the participants, including age, gender, marital status of the parents, occupation, education, field of study, an academic semester, university name, and living place. The 20-item spiritual well-being scale of Palutzian & Ellison [15] was used to measure the students' spiritual well-being, which consists of two subscales of religious well-being and existential well-being. The students were asked about the extent of their spiritual well-being on a six-point Likert scale, ranging from 1 (strongly disagree) to 6 (strongly agree). The score for spiritual well-being was the sum of the two subscales ranging from 20 to 120. The scores of 20–40 show low, 41–99 show moderate, and 100–120 show high spiritual well-being. The Persian version of the scale was tested for validity and reliability, and its validity and reliability have been confirmed (alpha coefficient = 0.85) [16].

### 2.4. Data Collection and Analysis

The researcher referred to research settings after obtaining the code of ethics and presented a written letter to the university's management. The researchers referred to libraries, study halls, student dormitories, and classes at Shahid Bahonar University and the University of Medical Sciences in Kerman in the morning and afternoon. The students completed and delivered the self-report questionnaires. The inclusion criteria were students of universities of Shahid Bahonar and Medical Sciences, who were willing to participate in this study. Guest students and incomplete questionnaires were excluded from the study.

Data were analyzed with SPSS version 25. Frequency, percentage, mean, and standard deviation were used to describe the studied variables. The independent *t*-test was used for the spiritual well-being score between the two groups. Independent *t*-test ANOVA, the Mann–Whitney *U*, and Kruskal–Wallis tests were used to compare the spiritual well-being score regarding demographic variables in each group. The significance level considered <0.05.

### 2.5. Ethical Consideration

The Kerman University of Medical Sciences approved this project (IR.KMU.REC.1396.1798). Then, permission was issued to the management of the two universities. Some oral information was given to the participants, including the goals and objectives of the study and the confidentiality and anonymity of the data, and that they were free to withdraw from the study at any time. Informed consent was obtained from all individual participants included in the study.

## 3. Results

### 3.1. Sociodemographic Characteristics

Six hundred and twenty-six students (313 nonmedical science and 313 medical science students of Kerman universities) participated in this study. The mean ages of nonhedical science and medical science students were 20.87 ± 3.62 and 21.81 ± 4.03, respectively. 61 percent of the nonmedical science students and 73.8 percent of the medical science students were females, and 88.2 percent of the medical science students and 88.7 percent of the nonmedical science students were single. In addition, 11.8% of the nonmedical science and medical science students were employed, and 99.7% of the nonmedical science students and 68.4% of the medical science students had associate and bachelor's degrees (Table 1).

### 3.2. The Comparison of Spiritual Well-Being between Medical and Nonmedical Science Students

The mean spiritual well-being of the nonmedical science students was 89.83 ± 16.79, and it was 81.61 ± 15.21 in the medical science students. A significant difference was observed between nonmedical science and medical science students in the scores of spiritual well-being and its two components, and the spiritual well-being of the nonmedical science students was higher than that of the medical science students. Furthermore, 1% of the nonmedical science students (*n* = 3) had a low level, 67.7% (*n* = 212) had a moderate level, and 31.3% (*n* = 98) had a high level of spiritual well-being. 0.3% of the medical science students (*n* = 1) had a low level, 84% (*n* = 263) had a moderate level, and 15.7% (*n* = 49) had a high level of spiritual well-being (Table 2).

### 3.3. The Spiritual Well-Being according to Sociodemographic Subcategories of Students

There was no correlation between age and spiritual health (*r* = 0.08, *p*=0.87). In addition, females had higher spiritual health than males. A significant association was observed between spiritual well-being and academic year, and junior students had significantly higher spiritual health than the first year (*p*=0.007), the ≥ fifth year (*p*=0.04). In medical science university students, no correlation was found between age and spiritual health (*r* = 0.02, *p*=0.73). Students who were in the third and fourth years of their educational years had higher spiritual health than students who were in the first and second years of their education (*p*=0.001). In nonmedical science universities, no correlation was found between age and spiritual health (*r* = 0.02, *p*=0.72). Also, females had higher spiritual health than males. In nonmedical sciences universities, no association was found between spiritual well-being and educational year (Table 3).

## 4. Discussion

The mean spiritual well-being of the nonmedical science students was 89.83 ± 16.79, and that of medical students was 81.61 ± 15.21, which was consistent with the results of Febbris et al. [5]. They acknowledged that students in this phase of their lives had to cope with this new situation, so spirituality could be an important coping strategy and protect against insecurity [5]. Feizi et al. in Iran also showed a moderate amount of spiritual well-being of students. Owing to the fact that spirituality and spiritual values have a special place in Islam and help individuals achieve inner peace and increase their ability to effectively manage stress, such findings are somewhat expected for Iranian people [11].

Spiritual well-being and its dimensions (existential well-being and religious well-being) were significantly higher in the nonmedical science students than the medical science students. In the present study, the difference between medical and nonmedical students about spiritual well-being could be clinically significant as it is obvious by the amount of effect size, i.e., 51. These results were consistent with the results of Yang et al. [17] and inconsistent with the results of Al-Qahtani. Their results showed better health responsibility and spiritual growth in the medical students than the nonmedical group and said it is necessary to introduce a well-being and health curriculum and a culture that promotes spiritual well-being [3]. Joys and Yanzom and Tapley et al. [18] revealed no significant difference between medical and nonmedical professional students in spiritual growth [19]. The gender differences with religious and cultural factors may be the reason for different results.

The results showed that the nonmedical science female students had more spiritual well-being than the male students, which was supported by Joys and Yanzom [19]. These results may be due to different social customs, life experiences, coping strategies, the various roles and characteristics of women, and their greater consistency with the spiritual principles. However, this difference was not observed among medical science students. These results were consistent with the results of Haghighat and Koolaee [20], who found no significant difference in spiritual well-being among male and female nurses. They believed that the spiritual well-being of the nurses caring for patients in medical wards was one of the effective psychological variables and special attention should be paid to spiritual well-being in the education of nurses. However, these results were inconsistent with the results of Tavan et al. [21], Rahimi et al. [6], and Ziapour et al. [22]. They demonstrated that the roles, characteristics, and behaviors socially attributed to women might be more consistent with some of the religious and spiritual principles and norms.

In this study, the spiritual well-being of married and single individuals was not different among medical and nonmedical science students which is consistent with the studies of Rahimi et al. (2014) [6] and Papazisis et al. [10] and inconsistent with the results of Tavan et al. (2015) [21] and Najarkolae et al. [7]. Furthermore, Soleimani et al. [23] concluded that singleness was associated with less spiritual well-being compared with marriage. They argued that spirituality was a critical and positive component in marital life, and people with high spiritual well-being were more committed to their spouses and considered such a commitment as a spiritual purpose.

The results of this study showed that the spiritual well-being of the medical and nonmedical students was not different regarding location, which was not supported by Tavan et al. [24]. They noticed that total spiritual well-being scores of nursing students not living in dormitories were higher than those of the students living in dormitories because they were living with their parents and did not experience problems of being away from home and family promoted spirituality in students.

The results of this study showed that the third-year medical science students had higher spiritual well-being than others, which supported the results of Al-Qahtani [3] and did not support the results of Rahimi et al. [6]. However, this difference was only observed in medical science students and was not observed among nonmedical science students. Farahaninia et al. found that by understanding the patient's spiritual care along with physical care, medical science students would clinically enjoy greater spiritual well-being and perform medical and nursing care and also there was a real relationship between spiritual care and inner spirituality, meaning that the stronger and higher the inner spirituality of the nurse, the more often he/she will try to provide spiritual care to the patient [25].

The results also showed no significant relationship between age and spiritual well-being among medical and nonmedical science students, which was inconsistent with the studies of Rahimi et al. [6] and Tavan et al. [21]. Ziapour et al. [22] revealed that the mean scores of spiritual well-being were significantly different in various ages, and the older the age, the higher the students' spiritual well-being. Yuen believed that spirituality had a direct relationship with aging when encountering the reality of death and getting adapted to it [26]. Differences in the value and atmosphere of spirituality in the studied cities may be the reasons for the contradiction of the studies mentioned above.

In this study, self-reporting was used to collect data, so students might not provide accurate information. There are limitations in terms of generalizations, interpretations, and etymological variables because of students' cognitive, social, and familial differences. Therefore, it is suggested that further research be conducted on statistical populations with other sociofamilial situations. In addition, the students' disciplines at the Shahid Bahonar University were very diverse; however, only 2% (*n* = 6) of them were studying Quranic sciences disciplines. Therefore, it is suggested that further research be conducted on students with different religious studies disciplines.

## 5. Conclusion

The results showed a statistically significant difference between medical and nonmedical science students in the scores of spiritual well-being and its two components, and the spiritual well-being of the nonmedical science students was higher than that of the medical sciences ones. It can be concluded that paying attention to young people's demographic characteristics, such as gender, marital status, housing status, and university type, can be useful in the enhancement of spiritual well-being because spirituality is important for the profession of medical science students, so educational programs may be necessary to expand this component in their curriculum. Regarding the lack of research on factors predicting spiritual well-being in the youth group, especially students, it is suggested that this issue be investigated in other provinces for the promotion of spiritual well-being.

## Figures and Tables

**Table 1:** Sociodemographic characteristics of the sample (*n* = 626).

Variable	Medical science students		Nonmedical science students	
	Mean	SD	Mean	SD
Age (yr.)	20.87	3.62	21.81	4.03
Variable	Frequency	Valid percent	Frequency	Valid percent
*Educational year*				
First	122	39	103	32.9
Second	83	26.5	92	29.4
Third	54	17.3	62	19.8
Forth	29	9.3	31	9.9
Fifth and upper	25	8	25	8
*Sex*				
Female	189	61	231	73.8
Male	121	39	82	26.2
*Marital status*				
Single	272	87.7	272	87.2
Married	38	12.3	40	12.8
*Occupation*				
Unemployed	276	88.2	269	88.2
Employed	37	11.8	36	11.8
*Living with both parents*				
Yes	280	90.3	282	93.4
No	30	9.6	20	6.6
*Educational grade*				
Bachelor's degree	312	99.7	214	68.4
Master's degree/professional doctorate	1	0.3	99	31.6
*Major*				
Medical science	3	1	313	100
Nonmedical science	310	99	0	0
*Living place*				
Student dormitory	115	38.2	204	65.4
Rented house	26	8.6	13	4.2
Resident of Kerman	160	53.2	95	30.4

In cases that the frequency was less than 313, there were missing values.

**Table 2:** Comparison of scores of spiritual well-being and its dimensions among nonmedical and medical science students.

Variable	Nonmedical science students		Medical science students		Independent *t*-test	*p* value	Effect size
	Mean	SD	Mean	SD			
Spiritual well-being	89.83	16.79	81.61	15.21	6.42	<0.001	0.51
Religious well-being	48.10	9.34	42.08	8.21	8.59	<0.001	0.68
Existential well-being	41.71	9.36	39.49	7.90	3.20	0.01	0.26

**Table 3:** Scores of spiritual well-being regarding demographic variables of students.

Variable	Spiritual well-being (total sample)		Statistical test and *p* value	Spiritual well-being (nonmedical science students)		Statistical test & *p* value	Spiritual well-being (medical science students)		Statistical test and *p* value
	Mean	SD		Mean	SD		Mean	SD	
*Educational year*									
First	84.03	15.45	*H* = 13.36 *p*=0.01	89.51	16.10	*F* = 1.14 *p*=0.34	77.53	11.76	*H* = 24.29 *p* < 0.001
Second	85.19	16.02		91.61	16.37		79.39	13.32	
Third	90.44	18.52		91.67	18.02		89.37	19.02	
Forth	87.22	17.71		86.52	18.55		87.87	17.16	
Fifth and upper	82.44	14.76		85.32	16.47		79.56	12.50	
*Sex*									
Female	86.85	15.86	*t* = 2.43	92.52	15.22	*t* = 3.48	82.21	14.87	*t* = 1.18
Male	83.42	17.74	*p*=0.01	85.80	18.46	*p*=0.001	79.91	16.10	*p*=0.24
*Marital status*									
Single	85.39	16.72	*t* = −1.37	89.40	17.22	*t* = −1.22	81.38	15.21	*t* = −0.83
Married	88.13	15.21	*p*=0.17	92.97	13.70	*p*=0.22	83.52	15.29	*p*=0.41
*Occupation*									
Unemployed	85.53	16.73	*t* = −1.48	89.41	17.22	*Z* = −1.13	81.56	15.26	*t* = −0.93
Employed	88.57	14.85	*p*=0.14	92.95	12.96	*p*=0.26	84.08	15.49	*p*=0.35
*Living with both parents*									
Yes	85.74	16.72	*t* = 0.45	89.85	16.49	*t* = 0.45	81.67	15.49	*Z* = −0.24
No	84.64	13.71	*p*=0.45	88.40	15.46	*p*=0.65	79.0	7.99	*p*=0.67
*Living place*									
Student dormitory	84.55	16.64	*F* = 1.70 *p*=0.18	90.83	16.89	*F* = 0.30 *p*=0.74	81.02	15.45	*F* = 1.30 *p*=0.30
Rented house	86.18	20.28		90.35	21.54		77.85	14.92	
Resident of Kerman	15.86	0.10		89.24	16.18		83.52	14.69	

*t* = independent *t*-test, *F* = analysis of variance, *Z* = Mann–Whitney *U* test, *H* = Kruskal–Wallis.

## Data Availability

Data are available upon request to the corresponding author via email.
